# Which Factor Is More Relevant to the Effectiveness of the Cognitive Intervention? A Meta-Analysis of Randomized Controlled Trials of Cognitive Training on Symptoms and Executive Function Behaviors of Children With Attention Deficit Hyperactivity Disorder

**DOI:** 10.3389/fpsyg.2021.810298

**Published:** 2022-01-13

**Authors:** Shuxian Chen, Jinglong Yu, Qiang Zhang, Jin Zhang, Ying Zhang, Junhong Wang

**Affiliations:** ^1^Foshan Hospital of Traditional Chinese Medicine, Foshan, China; ^2^Dongzhimen Hospital, Beijing University of Chinese Medicine, Beijing, China; ^3^The Third Affiliated Hospital of Sun Yat-sen University, Guangzhou, China; ^4^Center for Evidence Based Chinese Medicine, Beijing University of Chinese Medicine, Beijing, China

**Keywords:** attention deficit disorder with hyperactivity, cognitive training, executive function (EF), meta-analysis, children

## Abstract

**Objective:** Attention deficit hyperactivity disorder (ADHD) is a neurodevelopmental disorder characterized by developmentally inappropriate inattention, hyperactivity, and impulsivity. Multiple cognitive training appeared to be more effective than working memory training, but the evidence remains insufficient, particularly for the subgroup symptoms and executive function behaviors at home. Further analysis of the impact of factors on the effectiveness would facilitate the development of cognitive training.

**Methods:** We searched PubMed, Cochrane Library, Psyche, Embase, Chinese Biomedical Literature Database, CNKI, and Weifang Database, and included randomized controlled trials (RCTs) of children with ADHD undergoing cognitive intervention. Metaanalysis and univariate metaregression were performed by STATE. The risk of bias was assessed with the Cochrane risk of bias tool 2.0 by the two investigators separately. This study was registered with INPLASY, number INPLASY202140065.

**Results:** We included 17 RCTs in the systematic review, with a combined 1,075 participants. For metaanalyses of both subgroups of ADHD symptoms and the executive function behaviors, the test of published bias failed to reach the *p* < 0.05 level. When all of the training are considered together, cognitive training can improve the presentation of inattention symptoms [SMD = −0.390, 95%CI (−0.675, −0.104)] and executive function behaviors (SMD = −0.319, 95%CI (−0.527, −0.111)]. In the subgroup analysis, the effects of working memory training on both presentations were not statistically significant. In contrast, the multiple cognitive training had significant effects on the presentation of inattention symptoms [SMD = −0.507, 95% CI (−0.722, −0.292)], hyperactivity/impulsivity [SMD = −0.305, 95% CI (−0.518, −0.09)], and the executive function behaviors [SMD = −0.499, 95%CI (−0.707, −0.290)]. In addition, metaregression analysis showed that only training frequency did significantly impact the symptoms of ADHD and the executive function behaviors.

**Conclusion:** This study showed that improvements in symptoms and executive function behaviors were related to the domains of cognitive intervention. The findings suggest that multiple domains of cognitive training and moderate training frequency may have wider clinical benefits. All the above results highlight further research in refining the executive functions of children with ADHD and developing individually tailored cognitive intervention on homes based for children with vulnerable executive functions.

**Systematic Review Registration:** [http://inplasy.com/], [INPLASY202140065].

## Introduction

Attention deficit hyperactivity disorder (ADHD) is a neurodevelopmental disorder characterized by developmentally inappropriate inattention, hyperactivity, and impulsivity (Gallo and Posner, 2016). The impairing condition in children with ADHD profoundly affects academic performance, social interactions, and well-being (Wolraich et al., 2020). In the literature, the worldwide prevalence of ADHD was 7.2% in children and adolescents (Thomas et al., 2015). A systematic review estimated that the pooled prevalence of ADHD among children and adolescents in China was 6.26% (Wang et al., 2017). A wide variety of approaches have been used for the treatment of ADHD, including pharmacological and psychological interventions, parental practices, and dietary management. Although medication-based treatments for ADHD are currently widely used, psychostimulants are recommended as first-line treatment to manage ADHD symptoms in most guidelines (Posner et al., 2020). Still, it has several areas of concern with stimulant medications that are worth further consideration. For like emergent or early adverse events, psychiatric symptoms were associated with the medicines (Wigal, 2009); partial or poor response (Cerrillo-Urbina et al., 2018); intolerance of initiating treatment and triggering additional antianxiety or antidepressant treatments (Biederman et al., 2021); and the potential risk of stimulant misuse and diversion (Chang et al., 2014). Due to the limitations of medication, children with ADHD need more alternative and accessible therapies.

With the extensive body of research into the etiology and pathophysiology of neurodevelopmental disorders, such as ADHD and autism, more neuropsychological mechanisms could predict behavioral performance and support the design of non-pharmacological therapies (Hoogman et al., 2020; Wadhera and Kakkar, 2020). Many studies have found that ADHD may be associated with deficits in a variety of cognitive domains. Cognitive training, such as executive function training, that could directly target the multiple neuropsychological domains, may benefit children with ADHD. Laboratory studies have found accumulative evidence of deficits in executive functions such as behavioral inhibition, working memory, set-shifting, and planning and organization in groups of individuals with ADHD compared with non-affected controls (Brown, 2008). While multiple cognitive training can improve total ADHD symptoms compared with working memory training, according to previous findings, there are limited quantitative evaluations of multiple cognitive training on both symptoms of ADHD and executive function behaviors rated by parents. Currently, due to the impact of the novel coronavirus epidemic, teletherapy and rehabilitation based on the home environment have received widespread attention from physicians and patients. Some scholars have suggested that assistive technology-based interventions may improve the quality of life and psychological well-being for people with neurodegenerative diseases, thereby reducing feelings of isolation and improving their quality of life and psychological well-being (Matamala-Gomez et al., 2021). This inspired us to focus on the way cognitive interventions are delivered and the context in which they are applied.

The aim of this metaanalysis and systematic review was to assess the effect of cognitive intervention on symptoms and executive function behaviors of children with ADHD.

## Materials and Methods

### Selection Criteria and Literature Search

Selection criteria were identified according to the PICO principle. Studies were included if they conformed to the following inclusion and exclusion criteria. The intervention consisted of cognitive training or executive function training targeting domains of neuropsychological deficit (e.g., working memory, attention, inhibitory control, and cognitive flexibility, etc.). The control conditions were treated as usual, waiting list, active/placebo/sham (i.e., involving other computer-based) activities, or alternative training programs. Outcomes included the presentation of ADHD symptoms (inattentive and hyperactive/impulsive symptoms) and parent ratings of executive function behaviors [e.g., Behavior Rating Inventory of Executive Function (BRIEF)]. The BRIEF includes global executive composite (GEC) index, behavioral regulation index (BRI), and metacognition index (MI), derived from eight general executive function subscales, exploiting the efficacy of gathering structured observations of executive functions in daily life environments (Gioia et al., 2002; Isquith et al., 2013). Study types were limited to randomized controlled trials. Articles written in languages other than English were eligible. Participants were between 3 and 18 years of age. They met valid diagnostic criteria for ADHD, including the American Diagnostic and Statistical Manual of Mental Disorders 4th or 5th editions, the International Classification of Diseases 10th edition, and the Chinese Guidelines for the Prevention and Treatment of Attention Deficit Hyperactivity Disorder 2nd edition. Children were excluded if they had comorbidities with pervasive developmental disorders and severe psychiatric diagnoses that would prevent them from participating in treatment, other chronic medical/neurological conditions, intellectual disability with an estimated intelligence quotient < 70, or involved in other non-pharmacological treatment for ADHD. Articles not excluded after the title and abstract screening were obtained in full text and further evaluated against the exclusion criteria by two independent investigators.

English literature databases (Embase, PubMed, Cochrane library, and Psycnet) were systematically searched using predefined terms based on Sonuga-Barke et al. until August 2021. Wanfang Data Knowledge Service Platform, China National Knowledge Infrastructure (CNKI), and SinoMed as Chinese databases were searched following the same strategy as the former. The following mesh terms were used: attention deficit disorder with hyperactivity, cognitive training, randomized controlled trial, and executive function training. Detailed information on the search strategy and syntax for each database is available in “Supplementary Appendix 1.”

### Data Extraction and Statistical Analysis

Two review authors had independently extracted data using specially developed forms based on the clinical research elements. The risk of bias of included studies that were assessed with the Cochrane risk of bias tool 2.0 by two investigators separately, in terms of five domains of the Cochrane Collaborations tool: namely selection bias, performance bias, detection bias, attrition bias, and other bias. Any disagreement was again resolved by consensus with the senior authors. Data were exported to STATA Version 14 statistical software for analysis. The effect size was calculated using the random-effect model for each variable by the reported data (mean, standard deviations, and sample size). SMD was calculated as the mean of post-treatment in the intervention group minus the mean of post-treatment in the control group divided by the pooled pre-test standard deviation with a bias adjustment. Given the inherent heterogeneity of studies, the random effect model was used. To evaluate the possible sources of heterogeneity, subgroup analyses were conducted. When significant heterogeneity was observed, sensitivity analyses were performed. For all analyses, significance was determined by *p* < 0.05. The *I*^2^ was calculated *a posteriori* to estimate between-trial SMD heterogeneity. Publication bias was assessed with funnel plots and Egger’s tests. Univariate residual maximum likelihood (REML)-based meta-regression analyses on presentation and behaviors were used to assess the effect of potential factors of training (e.g., sessions, frequency, combination of medication, and duration).

## Results

### Study Characteristics and Assessment of Risk of Bias

**FIGURE 1:**
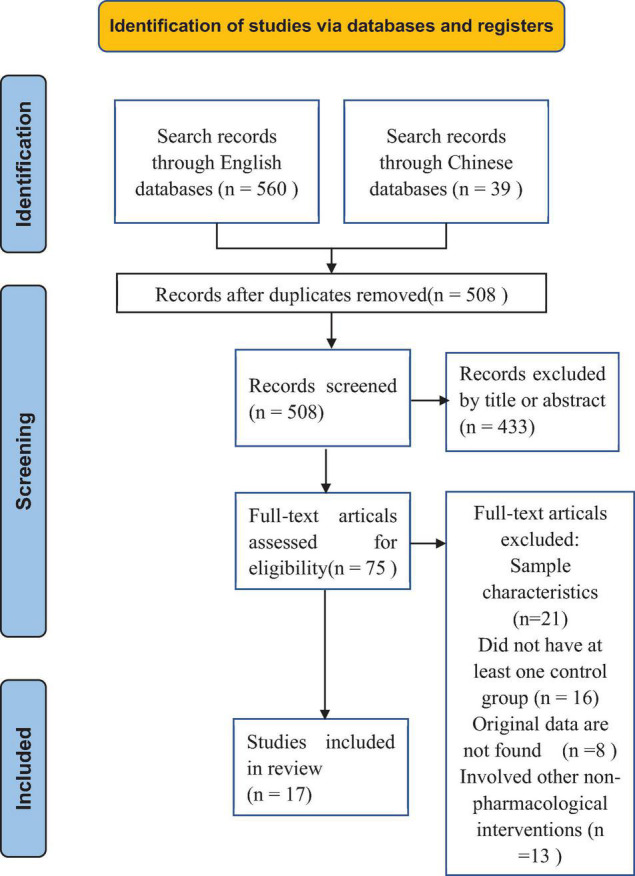
PRISMA flow diagram of selection of studies.

Table 1 summarizes the characteristics of 17 included studies. Figure 1 illustrates the selection process in the PRISMA flowchart. The review included 1,075 individuals, and effect estimates were based on 904 participants. The risk of bias for three trials is assessed as low risk. Eleven trials were scored as high risk, and three trials scored as some concerns. The details for the risk of bias assessment are depicted in “Supplementary Appendix 2.” Seven studies from America, seven from Europe, and three from Asia were included.

**TABLE 1:** Characteristics of the studies included in the analysis.

Studies	Training (*n*)/Control (*n*)	Setting	Age	Medication (%)	Criteria used for diagnostic	Outcomes	Duration (weeks) and sessions	Compliance (%)	Risk of bias
Steiner et al., 2011	MCT (8)/Waitlist (13)	School	12.4 ± 0.9	60	Not given	CRS-R; BRIEF	16/32	93	H
Egeland et al., 2013	WMT (33)/TAU (34)	School	10.4 ± 0.0.7	61	ICD-10	ADHD–RS; BRIEF	7/25	97	H
Tamm et al., 2013	Attention (54)/Waitlist (51)	Home	9.3 ± 1.4	68.6	DSM-4	SNAP- IV; BRIEF	8/16	97	H
van Dongen-Boomsma et al., 2014	WMT (26)/Placebo (21)	Home	6.5 ± 0.6	0	DSM-IV-TR	ADHD-RS; BRIEF	5/25	85	H
Chacko et al., 2014	WMT (44)/Placebo (41)	Home	8.4 ± 1.4	29.4	Kiddie-SADS	DBDR	5/25	78	H
Steiner et al., 2014	MCT (34)/Waitlist (36)	School	8.9 ± 1.0	48.6	DSM-4	Conners 3-P	20/40	97	S
van der Oord et al., 2014	MCT (18)/Waitlist (22)	Home	9.79 ± 1.04	72.5	DSM-IV	DBDRS; BRIEF	6/25	93	H
Tamm and Nakonezny, 2015	MCT (10)/Waitlist (9)	Home	5.0 ± 1.3	0	DSM-5	SNAP-IV; BRIEF	8/8	76	H
Dovis et al., 2015	MCT (30) Braingame Brian (BGB)/Placebo (30)	Home	10.6 ± 1.4	72.4	DSM-IV-TR	DBDRS; BRIEF	5/25	95	L
Bigorra et al., 2016	WMT (27)/Placebo (27)	Home	8.79 ± 1.75	0	DSM-IV-TR	BRIEF	5/25	94	H
Steeger et al., 2016	WMT (22)/Placebo (23)	Home	12.0 ± 1.0	65.2	DSM-IV	ADHD-RS; BRIEF	5/25	94	H
Azami et al., 2016	MCT (12)/Placebo (11)	Clinic	7–12	17.4	Not given	SNAP-IV	8/20	100	H
Qian et al., 2017	MCT (38)/Wait-list (30)	Clinic	8.3 ± 1.3	14.7	DSM-IV	ADHD-RS; BRIEF	12/12	79	H
Ackermann et al., 2018	WMT (18)/TAU (10)	Home	13.8 ± 0.9	100	DSM-IV	CRS-R	5/25	58	S
Rivard et al., 2020	WMT (17)/Placebo (14)	Home	7–13	54.8	DSM-5	Conners 3-P; BRIEF	6/25	60	S
Jones et al., 2020	MCT (41)/Active knowledge training (39)	Home	10.14 ± 2.02	Not given	DSM-IV	CPRS–R; BRIEF	4/20	79	H
Hahn-Markowitz et al., 2020	MCT (50)/Wait (51)	Clinic	8.5 ± 0.85	65.40%	DSM-IV	CPRS–R; BRIEF	12/12	94	L

*MCT, multiple cognitive training; WMT, working memory training; TAU, treatment as usual; DSM-IV-TR, Diagnostic and Statistical Manual of Mental Disorders (4th ed. text rev); ICD-10, The International Classification of Diseases the Tenth Revision; DSM-V, Diagnostic and Statistical Manual of Mental Disorders (5th ed. DSM-5); DSM-IV, Diagnostic and Statistical Manual of Mental Disorders (4th ed. DSM-IV); Kiddie-SADS, Kiddie Schedule for Affective Disorders and Schizophrenia; CRS-R, Conners’s Rating Scales–Revised; BRIEF, behavioral rating inventory of executive functioning; ADHD–RS, ADHD-Rating Scale; Conners 3-P, Conners-3 Parent; SNAP-IV, Swanson, Nolan, and Pelham DSM-IV, ADHD Rating Scale; DBDRS, Disruptive Behavior Disorder Rating scale; CPRS-R, Connors’ Parent Rating Scale revised.*

**FIGURE 2:**
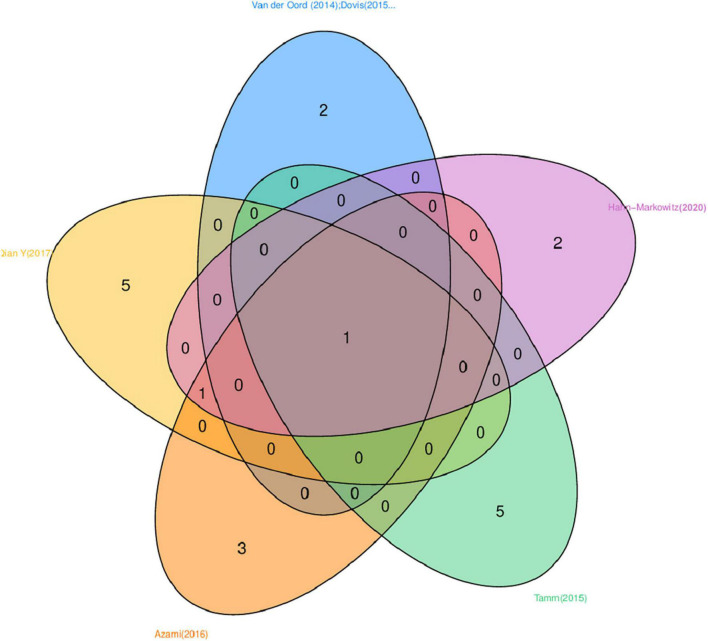
Venn diagram.

Seven trials used working memory training that involved computer-based programs (Egeland et al., 2013; Chacko et al., 2014; van Dongen-Boomsma et al., 2014; Bigorra et al., 2016; Steeger et al., 2016; Ackermann et al., 2018; Rivard et al., 2020); one trial involved attention training (Tamm et al., 2013); nine trials utilized multiple cognitive training (i.e., program targeting more than one neuropsychological domain); two used attention and working memory training (Steiner et al., 2011, 2014); one used inhibition and working memory training (Jones et al., 2020); two used working memory, inhibition, and cognitive flexibility (van der Oord et al., 2014; Dovis et al., 2015); one used effortful executive strategies (inhibition, effort, monitoring, and planning) (Hahn-Markowitz et al., 2020); one used inhibition, planning and time management, sustained attention, organization, cognitive flexibility, working memory (Qian et al., 2017); one involved practicing tasks specially designed to train selective, sustained and divided attention, interference inhibition (interference control), short-term memory, planning, and processing speed (Azami et al., 2016), and one trial provided a general executive function training covering attention, inhibition, memory, hand-eye coordination, balance, sensory awareness, and listening (Tamm and Nakonezny, 2015). Eight trails of multiple cognitive training included memory training and seven involved inhibition. Venn diagram visualization is used to display the overlapping domains in training involving at least three or more neuropsychological domains. As depicted in Figure 2, the five completely non-overlapping areas represent distinct areas within each trial, with the middle area of complete overlap showing the number “1” indicating that these studies share one domain, namely inhibition. In multiple cognitive training trials, six involved computerized training, and three used several practical activities and manualized performance tasks. Seven trials had a placebo control condition, in which schedules had an adaptive component; task difficulty was increased across sessions to track performance improvement. Seven trials had a waitlist control, one trial had an active knowledge training control, two trials had control groups without any training but receiving medication-continued routine treatment. Eleven trials were implemented at home, three at school, and three in the clinic. The adherence rate for all included studies is 87.1%, for working memory intervention groups it is 82.1%, and for multiple cognitive intervention groups it is 88.6%.

### Presentation of Attention Deficit Hyperactivity Disorder Symptoms

The first set of analyses examined the impact of cognitive training on the presentation of inattention and hyperactivity/impulsivity symptoms (SMD and CI data are presented in Table 2 and “Supplementary Appendix 3”; sensitivity analyses are presented in “Supplementary Appendix 4”). Fifteen trials reported outcomes and involved six rating instruments in assessing symptoms by parents or clinicians. Four trials of working memory training controlled with placebo (non-adaptive) training are probably blinded measures. Two of the multiple cognitive training trials used blinded measurements.

**TABLE 2:** Summary of results showing pooled standardized mean differences (SMD) between treatment and control arms for each outcome.

Outcome	subgroup	evaluator	Study *n*	Effect		Heterogeneity	
				SMD	95%CI	*I* ^2^	*P*
Inattention symptoms	Overall	Clinician- or parent-rated	15	–0.390	–0.675 to –0.104	71.4%	0.000
		Only parent-rated	13	–0.390	–0.699 to –0.080	73.8%	0.000
	Blind	Clinician- or parent-rated	6	–0.003	–0.335 to 0.329	46.7%	0.095
	Attention	Parent-rated	1	–1.460	–1.892 to –1.028	N	N
	WMT	Clinician- or parent-rated	6	0.042	–0.233 to 0.317	28.2%	0.224
		Only parent-rated	5	0.034	–0.300 to 0.368	42.4%	0.139
	MCT	Clinician- or parent-rated	8	–0.507	–0.722 to –0.292	0.0%	0.619
		Only parent-rated	7	–0.481	–0.702 to –0.261	0.0%	0.640
Hyperactivity/impulsivity symptoms	Overall	Clinician- or parent-rated	15	–0.160	–0.367 to 0.046	46.7%	0.024
		Parent-rated	13	–0.190	–0.400 to 0.020	44.5%	0.042
	Attention	Parent-rated	1	–0.569	–0.959 to –0.178	N	N
	WMT	Clinician- or parent-rated	6	0.127	–0.171 to 0.425	38.3%	0.151
		Parent-rated	5	0.061	–0.271 to 0.392	41.5%	0.144
	MCT	Clinician- or parent-rated	8	–0.305	–0.518 to –0.093	0.0%	0.588
		Parent-rated	7	–0.294	–0.513 to –0.076	0.0%	0.492
GEC of BRIEF	Overall	Parent-rated	13	–0.319	–0.527 to –0.111	44.5%	0.042
	Blind	Parent-rated	5	0.052	–0.252 to 0.356	10.7%	0.345
	Attention	Parent-rated	1	–0.821	–1.219 to –0.422	N	N
	WMT	Parent-rated	5	0.091	–0.163 to 0.345	0.0%	0.575
	MCT	Parent-rated	7	–0.499	–0.707 to –0.290	0.0%	0.997
BRI of BRIEF	Overall	Parent-rated	6	–0.182	–0.387 to 0.022	0.0%	0.439
	Attention	Parent-rated	1	–0.492	–0.881 to –0.104	N	N
	WMT	Parent-rated	3	0.049	–0.269 to 0.366	0.0%	0.948
	MCT	Parent-rated	2	–0.214	–0.582 to 0.155	0.0%	0.645
MI of BRIEF	Overall	Parent-rated	7	–0.244	–0.649 to 0.160	76.7%	0.000
	Attention	Parent-rated	1	–0.896	–1.298 to –0.494	N	N
	WMT	Parent-rated	3	0.254	–0.044 to 0.553	0.0%	0.501
	MCT	Parent-rated	3	–0.509	–0.831 to –0.187	0.0%	0.560

*GEC, global executive composite; BRI, behavioral regulation index; MI, metacognition index.*

When parents or clinicians reported outcomes, there was a moderate and significant effect on inattention, but no effect on hyperactivity/impulsivity. When analyses were set in blinded measures, effect sizes were not statistically significant for both symptoms. Between-study heterogeneity of overall effect sizes was high and significant. The subgroup analysis for SMD of each symptom found that the heterogeneity came from domains of training programs. When single and multiple domains of cognitive interventions stratified subgroup analyses, trials of multiple cognitive training revealed minor heterogeneity and showed a significant effect size for both presentations.

When analyses were restricted to outcomes reported by the parent, the effect size remained significant for inattention but not for hyperactivity/impulsivity. In subgroup analyses, trials of multiple cognitive training remained a significant effect size for inattention and hyperactivity/impulsivity symptoms. Despite the minor heterogeneity between trials of working memory training, the results have not yet shown significant benefits in any analysis. There was a lack of sufficient studies reported only by the parent (*n* < 5) for analysis of blinded measures.

### Behavior Rating Inventory of Executive Function

Thirteen trials included the assessment of executive function behaviors by BRIEF from parents. Five trials used working memory training, seven used multiple cognitive training, and only one trial on attention training (Table 2; sensitivity analyses are presented in “Supplementary Appendix 4”). The combined effect estimate with all interventions revealed a small significant effect size on the global executive composite (GEC) of the BRIEF. The heterogeneity was high, and the subgroup analysis revealed an explicit decrease in heterogeneity by stratified exposure interventions. These demonstrated a significant moderate effect size of multiple cognitive training on GEC of BRIEF. When analyses were set in blinded measures, effect sizes were not statistically significant.

Six studies reported the behavioral regulation index, and seven studies reported the metacognition index of the BRIEF. There were no significant effects on both indexes. Subgroup analysis similarly revealed a significant effect for multiple cognitive training.

### Metaregression

Potential factors related to the effect size were examined by univariate REML-based metaregression analyses. Table 3 shows that statistically significant effect size in both symptoms could be influenced by the frequency of training but not by sessions, durations, and combinations of medication. There were no significant effects in the global executive composite of BRIEF.

**TABLE 3:** Metaregression analysis based on REML.

Dependent	Covariates	Number of obs	Exp(b)	*P*
Inattention symptoms	Combination of medication	14	0.996	0.522
	Duration	15	0.966	0.273
	Sessions	15	1.026	0.197
	Frequency	15	1.108	0.017
Hyperactivity/impulsivity Symptoms	Combination of medication	14	0.996	0.367
	Duration	15	0.971	0.212
	Sessions	15	1.016	0.281
	Frequency	15	1.198	0.003
Global Executive Composite of BRIEF	Combination of medication	12	0.994	0.153
	Duration	13	0.977	0.334
	Sessions	13	1.014	0.281
	Frequency	13	1.027	0.056

### Publication Bias

Results of publication bias and Egger’s test are reported in Table 4 and “Supplementary Appendix 6.” For both presentation of ADHD symptoms and the global executive composite of the BRIEF, tests did not reach the *p* < 0.05 level.

**TABLE 4:** Publication bias with Egger’s test.

Small-study effects of publication bias	Number of studies	Coef	*t*	*P*	95%CI
Inattention symptoms	15	–1.231	–0.58	0.571	–5.808 to 3.345
Hyperactivity/impulsivity symptoms	15	–0.952	–0.62	0.544	–4.256 to 2.351
Global executive composite of BRIEF	13	0.558	0.33	0.745	–3.126 to 4.241

## Discussion

The results of our systematic review and metaanalysis indicated that cognitive training can alleviate the presentation of inattention and improve executive function behaviors in a parent-rated setting. The effect size should be considered convincing, but blind assessments were not enough. Therefore, the results were clinically uncertain. However, it also suggested that some interventions may prove better than others in most outcomes. To adjust and detect the reported heterogeneity of outcomes, we further applied subgroup analysis based on domains of training programs. Subgroup analyses indicated that working memory training does not benefit, whereas multiple cognitive training proved highly significant on ADHD symptoms and executive function behaviors.

The present work analyses the effects of working memory training on ADHD symptoms, which corresponds to an earlier review (Cortese et al., 2015). Our data also do not support the use of working memory training for reducing the presentation of inattention symptoms, neither hyperactivity/impulsivity symptoms. With an increasing number of randomized controlled trials containing multiple cognitive training (i.e., targeting more than one neuropsychological domain), there are sufficient trials to explore further the effects of multiple cognitive training on both presentations of ADHD, which only gave an effect for total ADHD symptoms in the previous metaanalysis (Cortese et al., 2015). In this work, the effect of multiple cognitive training is significant on the presentation of inattention symptoms in the parent-rated pattern. However, contrary to expectations, hyperactivity/impulsivity was not statistically significant. There are several possible explanations for this result. A regression model was employed to predict executive dysfunction based on both presentations of ADHD, and it found that inattention was significantly associated with executive functions weaknesses. Meanwhile, hyperactivity–impulsivity was not related to executive function performance independently when considering the impact of inattention, which suggests that inattention symptoms are more directly associated with neuropsychological impairment of ADHD rather than hyperactivity/impulsivity (Chhabildas et al., 2001). A metaanalysis on the DSM-IV subtypes indicated that executive function weaknesses are primarily directly associated with DSM-IV inattention rather than hyperactivity-impulsivity (Willcutt et al., 2005). It suggests that a substantial part of the impact of cognitive training on total ADHD symptoms comes from the contribution of relieving inattentive symptoms. Although the effect of cognitive training was significant in the GEC, it was not significant in the behavioral regulation index and metacognition index, possibly attributed to most of the single domain training programs included. Moreover, multiple cognitive training showed a better effect on the metacognition index than the behavioral regulation index in BRIEF. It proved significantly better than working memory, although still being a subgroup analysis. These findings indicate that the implementation of multiple cognitive interventions can improve inattention symptoms and executive functioning behaviors.

Though it makes intuitive sense to integrate an array of executive functions for the cognitive intervention of ADHD, many potential factors deserve discussion.

Regarding the first quantitative question on cognitive training, further statistics on the program and training compliance are presented in the results stratified by different domains of interventions. According to the characteristics of trials, working memory training programs are instituted for an average of 25 sessions, with 4.86 sessions per week, and 5.17 weeks of duration. Meanwhile, multiple cognitive training shows a longer duration (M. 11, s.d. 5) due to the more neuropsychological domains of executive function training involved. However, it is neither more frequent (2.94 per week) nor more sessions (M. 21.9, s.d. 9.69) than working memory training. It is interesting to note that in the metaregression analysis, we identified no significant effect of sessions and durations of cognitive training on any presentation of ADHD and the global executive composite of BRIEF. The most surprising aspect of the results is that training frequency significantly affects the SMD for each presentation.

Compliance was measured as the proportion of adhering participants. There are 2 two hypotheses for the treatment adherence of multiple cognitive training in ADHD. One of the issues that emerge from training programs is complexity, and long-term due to multiple training tasks may weaken adherence. On the contrary, the second hypothesis assumes that multiple cognitive interventions can improve compliance because treatment can reduce the boredom and monotony of single-task training for children with ADHD. This review shows a slightly higher compliance percentage to multiple cognitive training than to working memory training in both the intervention and control groups. This observation may support the hypothesis that the variety of cognitive training components may improve adherence to treatment for children with ADHD. The study of psychological time as information shows that the reduction of time promotes an increase of boredom and the under-motivation state, sometimes associated with a general decrease of well-being reflecting on adaptive behaviors (Zakay, 2014). These results suggest that the multiple tasks of cognitive training and adequate training frequency may be more accessible in translating the clinical benefits of ADHD than increasing training sessions.

Secondly, heterogeneity between cognitive training programs needs to be discussed. The finding that emerges from the subgroup analyses is that effects of multiple cognitive training have a higher level of homogeneity than working memory training. These findings suggest that a substantial part of the overall estimated cognitive training effect likely originates in the neuropsychologic heterogeneity of ADHD. Based on the previous work in this field, neurocognitive heterogeneity is gradually recognized as a pervasive phenomenon in ADHD (Nigg et al., 2005). The results of the present work may support the transition from models positing a single core deficit to multiple-deficit models, which represents a paradigm shift in the way that the neuropsychology of ADHD is conceptualized (Sonuga-Barke, 2003). ADHD has been considered a developmental impairment that relates to impaired executive function (EF) (Brown, 2008). Executive functions refer to interrelated, higher-order cognitive processes that enable goal-directed behavior and novel problem-solving strategies (Miyake et al., 2000). It consists of several domains: inhibition, initiation, sustaining attention, set-shifting, working memory, emotional regulation, planning, organizing, and monitoring (Castellanos et al., 2006). Multiple deficit models regard ADHD as the additive or interactive effects of dysfunction in multiple neural networks (Willcutt et al., 2005). According to Barkley (1997), ADHD arises from a primary deficit in a specific EF domain, such as response inhibition or working memory or more general weakness in executive control, which impedes maintaining attention to relevant cues while filtering irrelevant information (Barkley, 1997). Prior studies have noted the executive function theory of ADHD, which found that groups with ADHD exhibited significant impairment on most executive function tasks. A strong relationship between ADHD and significant weaknesses in several key EF domains has been reported in the literature, which was obtained on functions of response inhibition, vigilance, and working memory. Consistent with the report, this research found that the more common components of multiple cognitive training are working memory, inhibition, and attention. It should be noted that cognitive intervention would be able to pay broad attention to the training containing multiple executive functions, thereby enabling them to obtain more thorough intervention quality.

## Limitations

Several limitations need to be discussed when interpreting the current results. First, these promising findings are tempered by the limited number of trials (*n* = 6) that reported blind measurement. There were insufficient studies (*n* < 5) to analyze the effect of multiple cognitive training in blinded measures reported by parents. Therefore parents may have been affected by expectancy bias or the Hawthorne effect. Second, the use of a waitlist control group for participants who are more challenging to treat or who perceive the treatment as less beneficial may drop out of trials. However, the compliance was relatively reasonable in most trials. Third, it cannot be ignored that medications hold the first-line role in the treatment of ADHD. Exactly, many of the studies had inclusion criteria that allowed children with ADHD to maintain a well-adjusted and stable dose of medication intake, which reflected that cognitive training alone is less acceptable to children and guardians.

On the other hand, most parents remain open to non-pharmacological interventions, as evidenced by the overall proportion of adherence. In terms of metaregression analysis, we found no significant effect of medication on both presentation and the global executive composite of BRIEF. This finding is similar to the prior metaanalysis of the cognitive training effect that remained unaltered when the analysis controlled the no or low medication trials (Sonuga-Barke et al., 2013). Despite these problems, some of the included studies made qualified efforts to lessen the risk of bias judgment. A short discussion of the participant blinding quality assessments and allocation concealment is proposed. The domain-based tool was chosen as an assessment point in this review, which was recommended by the Cochrane collaboration with validation evidence in this realm. We believe that sufficient research has shown that cognitive training should be available to children and adolescence with ADHD.

## Conclusion

Cognitive training is a non-invasive, safe, and inexpensive intervention that can be implemented quickly and conveniently in a home setting. This metaanalysis aimed to synthesize more areas of populations, multiple cognitive training exposures, and parent-rated outcomes to augment the ecological validity while maintaining the suitability of evidence. In summary, multiple cognitive training alleviates the presentation of inattention and improves general executive function behaviors in children with ADHD. Even though people in some areas are quarantined at home because of the pandemic, in a way telemedicine can reduce concerns about the absence of medication or adverse effects caused by reliance on medication. Working memory training was proven ineffective in some cases, but it cannot be ignored as an essential executive function in line with previous studies. We advocate that multiple cognitive training should be available to compensate for the well-proven executive functions in neuropsychological domains rather than repeating them in a single-task model. That may benefit children with ADHD to coordinate various cognitive functions. Further research is needed to confirm and extend these psychology fields.

## Data Availability Statement

The original contributions presented in the study are included in the article/Supplementary Material, further inquiries can be directed to the corresponding author.

## Author Contributions

SC, JY, and JW contributed to the conception and design of the study. QZ and JZ screened the articles and extracted the data. JY performed the statistical analysis. SC wrote the first draft of the manuscript. JY and YZ wrote sections of the manuscript. All authors contributed to manuscript revision, read, and approved the submitted version.

## Conflict of Interest

The authors declare that the research was conducted in the absence of any commercial or financial relationships that could be construed as a potential conflict of interest.

## Publisher’s Note

All claims expressed in this article are solely those of the authors and do not necessarily represent those of their affiliated organizations, or those of the publisher, the editors and the reviewers. Any product that may be evaluated in this article, or claim that may be made by its manufacturer, is not guaranteed or endorsed by the publisher.
