# Supplementary material for: Which Factor Is More Relevant to the Effectiveness of the Cognitive Intervention? A Meta-Analysis of Randomized Controlled Trials of Cognitive Training on Symptoms and Executive Function Behaviors of Children With Attention Deficit Hyperactivity Disorder
Source: Front Psychol. 2022 Jan 13;12:810298. doi: 10.3389/fpsyg.2021.810298 (PMC8792444; doi:10.3389/fpsyg.2021.810298)
Supplement: Supplementary file 2 [file Data_Sheet_2.doc]

Appendix 1 Literature retrieval strategy

| Database | search strategy and syntax |
| --- | --- |
| China National Knowledge Infrastructure | （SU=' attention deficit disorder with hyperactivity ') AND （SU='cognitive training' + 'executive function training' + 'attention training' + 'working memory training'）AND（SU=' randomized controlled trial '） |
| Wanfang Data Knowledge Service Platform | theme:( attention deficit disorder with hyperactivity OR ADHD)*( cognitive training + executive function training + attention training + working memory training)*（randomized controlled trial） |
| SinoMed as Chinese databases | ( " attention deficit disorder with hyperactivity " OR " ADHD ") AND( "cognitive training" OR "executive function training" OR " working memory training" OR " attention training " )AND ("randomized controlled trial") |
| PubMed | ((((ADHD[Title/Abstract] OR adhd[Title/Abstract] OR attention deficit disorder with hyperactivity[Title/Abstract] OR minimal brain disorders[Title/Abstract] OR syndrome hyperkinetic[Title/Abstract] OR hyperkinetic syndrome[Title/Abstract] OR hyperactivity disorder[Title/Abstract] OR hyperactive child syndrome[Title/Abstract] OR childhood hyperkinetic syndrome[Title/Abstract] OR attention deficit hyperactivity disorders[Title/Abstract] OR attention deficit hyperactivity disorder[Title/Abstract] OR adhd attention deficit hyperactivity disorder[Title/Abstract] OR addh[Title/Abstract] OR overactive child syndrome[Title/Abstract] OR attention deficit hyperkinetic disorder[Title/Abstract] OR hyperkinetic disorder[Title/Abstract] OR attention deficit disorder hyperactivity[Title/Abstract] OR attention deficit disorders hyperactivity[Title/Abstract] OR child attention deficit disorder[Title/Abstract] OR hyperkinetic syndromes[Title/Abstract] OR syndromes hyperkinetic[Title/Abstract] OR hyperkinetic syndrome childhood)[Title/Abstract])) AND ((RCT[Title/Abstract] OR cluster RCT[Title/Abstract] OR clinical trial*[Title/Abstract] OR controlled clinical trial*[Title/Abstract] OR crossover procedure[Title/Abstract] OR cross over stud*[Title/Abstract] OR crossover design[Title/Abstract] OR double blind procedure[Title/Abstract] OR double blind method[Title/Abstract] OR double blind stud*[Title/Abstract] OR single blind procedure[Title/Abstract] OR single blind method[Title/Abstract] OR single blind stud*[Title/Abstract] OR random allocation[Title/Abstract] OR randomization[Title/Abstract] OR random assignment[Title/Abstract] OR randomized controlled trial*)[Title/Abstract])) AND ((cognitive training[Title/Abstract] OR attention training[Title/Abstract] OR working memory training[Title/Abstract] OR cognitive remediation[Title/Abstract] OR executive function training)[Title/Abstract]) |
| Embase | ('attention deficit disorder'/exp OR 'attention deficit disorder' OR 'hyperactive*':ti,ab,kw OR 'adhd*':ti,ab,kw OR 'hyperkine*':ti,ab,kw) AND ('random*':ab,ti OR 'placebo':ab,kw OR 'double-blind':ab,kw) AND ('cognitive training':ab,ti OR 'attention training':ab,ti OR 'working memory training':ab,ti OR 'cognitive remediation':ab,ti OR 'executive function training':ab,ti) AND ([child]/lim OR [adolescent]/lim) |
| Psycnet | Abstract: ADHD OR Abstract: ADDH OR Abstract: hyperkine* OR Abstract: hyperactive* OR Abstract: attention deficit* AND Abstract: cognitive training OR Abstract: working memory OR Abstract: attention training OR Abstract: executive function training OR Abstract: cognitive remediation AND Any Field: random* OR Any Field: placebo OR Any Field: blind AND APA Full-Text Only |
| the Cochrane Library | #1 MeSH descriptor: [Attention Deficit Disorder with Hyperactivity] this term only  #2 (‘ADHD’ OR 'ADDH'):ti,ab,kw OR ('attention deficit*'):ti,ab,kw OR ('hyperactive*'):ti,ab,kw OR ('hyperkine*'):ti,ab,kw (Word variations have been searched)  #3 ('cognitive training'):ti,ab,kw OR ('working memory training'):ti,ab,kw OR ('cognitive remediation'):ti,ab,kw OR ('executive function training'):ti,ab,kw OR ('attention training'):ti,ab,kw (Word variations have been searched)  #4 ('random*'):ti,ab,kw OR ('placebo'):ti,ab,kw OR ('double-blind'):ti,ab,kw (Word variations have been searched)  #5 (#1 OR #2) AND #3 AND #4 |
| *SU=subject;’TI=title;; ab=abstract；kw= keywords and keywords plus. | |

Appendix 2 Graphic output of the Cochrane risk of bias

| Study ID | Randomization process | Missing outcome data | Measurement of the outcome | Selection of the reported result | Selection of the reported result | Overall |
| --- | --- | --- | --- | --- | --- | --- |
| Van Dongen Boomsma(2014) | **+** | **+** | **+** | **+** | **！** | **+** |
| Steiner（2014） | **！** | **+** | **+** | **+** | **！** | **！** |
| Dovis（2015） | **！** | **+** | **+** | **+** | **+** | **+** |
| Chacko (2014) | **+** | **+** | **？** | **+** | **！** | **？** |
| Azami(2016) | **+** | **+** | **！** | **？** | **+** | **？** |
| Steiner (2011) | **！** | **！** | **+** | **？** | **！** | **？** |
| Egeland (2013） | **！** | **+** | **！** | **？** | **！** | **？** |
| Van der Oord (2014) | **+** | **！** | **+** | **？** | **！** | **？** |
| Tamm（2015） | **！** | **？** | **+** | **？** | **！** | **？** |
| Bigorra（2016） | **+** | **+** | **？** | **+** | **！** | **？** |
| Steeger（2016） | **！** | **？** | **？** | **！** | **！** | **？** |
| Qian Y（2017） | **+** | **！** | **？** | **？** | **！** | **？** |
| Tamm（2013） | **！** | **！** | **？** | **！** | **！** | **？** |
| Ackermann（2018） | **！** | **！** | **+** | **！** | **！** | **！** |
| Rivard（2020） | **！** | **！** | **？** | **+** | **！** | **！** |
| Jones(2020) | **！** | **？** | **？** | **+** | **？** | **？** |
| Hahn-Markowitz (2020) | **+** | **+** | **+** | **+** | **+** | **+** |

Appendix 3 Forest plots for meta-analysis of effects on symptoms of ADHD and Behavior Rating Inventory of Executive Function (BRIEF) assessed by parents or clinicians

Figure 1 Meta-analysis of effects on Inattention symptoms- reported by parents or clinicians

Figure 2 Meta-analysis of effects on Inattention symptoms - reported only by parents

Figure 3 Meta-analysis of effects on Inattention symptoms in blinded measures

Figure 4 Meta-analysis of effects on Hyperactivity/Impulsivity symptoms- reported by parents or clinicians

Figure 5 Meta-analysis of effects on Hyperactivity/Impulsivity Symptoms- reported only by parents

Figure 6 Meta-analysis of effects on Hyperactivity/Impulsivity Symptoms in blinded measures

Figure 7 Meta-analysis of effects on Global Executive Composite (GEC) of the BRIEF

Figure 8 Meta-analysis of effects on Global Executive Composite (GEC) of the BRIEF in Blind measurement

Figure 9 Meta-analysis of effects on Behavioral Regulation Index of the BRIEF

Figure 10 Meta-analysis of effects on Metacognition Index of the BRIEF

Appendix 4 Sensitivity Analyses

Figure 11 Sensitivity Analyses of effects on Inattention symptoms

Figure 12 Sensitivity Analyses of effects on Hyperactivity/Impulsivity symptoms

Figure 13 Sensitivity Analyses of effects on Global Executive Composite (GEC) of the BRIEF

Appendix 5 Bubble Charts of the Meta-Regression Analysis to Assess the Relationship Between Training Sessions, Durations, Frequency, Combination of Medication and Standardized Mean Difference (SMD) for both Symptoms of ADHD and Global Executive Composite of BRIEF

Figure 14 Relationship Between Combination of Medication and Standardized Mean Difference (SMD) for Inattention Symptoms

Figure 15 Relationship Between Training Duration and Standardized Mean Difference (SMD) for Inattention Symptoms

Figure 16 Relationship Between Training Sessions and Standardized Mean Difference (SMD) for Inattention Symptoms

Figure 17 Relationship Between Training Frequency and Standardized Mean Difference (SMD) for Inattention Symptoms

Figure 18 Relationship Between Combination of Medication and Standardized Mean Difference (SMD) for Hyperactivity/Impulsivity Symptoms

Figure 19 Relationship Between Training Duration and Standardized Mean Difference (SMD) for Hyperactivity/Impulsivity Symptoms

Figure 20 Relationship Between Training Sessions and Standardized Mean Difference (SMD) for Hyperactivity/Impulsivity Symptoms

Figure 21 Relationship Between Training Frequency and Standardized Mean Difference (SMD) for Hyperactivity/Impulsivity Symptoms

Figure 22 Relationship Between Combination of Medication and Standardized Mean Difference (SMD) for Global Executive Composite of BRIEF

Figure 23 Relationship Between Training Duration and Standardized Mean Difference (SMD) for Global Executive Composite of BRIEF

Figure 24 Relationship Between Training Sessions and Standardized Mean Difference (SMD) for Global Executive Composite of BRIEF

Figure 25 Relationship Between Training Frequency and Standardized Mean Difference (SMD) for Global Executive Composite of BRIEF

Appendix 6 Funnel Plots and Egger’s Tests for Meta-Analyses of Effects of Cognitive Training on Symptoms of Attention-Deficit/Hyperactivity Disorder (ADHD) and Global Executive Composite of BRIEF

Figure 26 Funnel Plots for Meta-Analyses of Effects of Cognitive Training on Inattention Symptoms

Egger's test for small-study effects:

Regress standard normal deviate of intervention

effect estimate against its standard error

Number of studies =15 Root MSE = 1.916

----------------------------------------------------------------------------------------------------------

Std_Eff | Coef. Std. Err. t P>|t| [95% Conf. Interval]

----------------------------------------------------------------------------------------------------------

slope | -.0127306 .6201681 -0.02 0.984 -1.352522 1.327061

bias | -1.231483 2.118432 -0.58 0.571 -5.808077 3.345111

---------------------------------------------------------------------------------------------------------

Test of H0: no small-study effects P = 0.571

Figure 27 Egger’s Tests for Meta-Analyses of Effects of Cognitive Training on Inattention Symptoms

Figure 28 Funnel Plots for Meta-Analyses of Effects of Cognitive Training on Hyperactivity/Impulsivity Symptoms

Egger's test for small-study effects:

Regress standard normal deviate of intervention

effect estimate against its standard error

Number of studies =15 Root MSE = 1.401

----------------------------------------------------------------------------------------------------------

Std_Eff | Coef. Std. Err. t P>|t| [95% Conf. Interval]

----------------------------------------------------------------------------------------------------------

slope | .1167271 .4409046 0.26 0.795 -.8357892 1.069244

bias | -.9522257 1.529125 -0.62 0.544 -4.2557 2.351248

----------------------------------------------------------------------------------------------------------

Test of H0: no small-study effects P = 0.544

Figure 29 Egger’s Tests for Meta-Analyses of Effects of Cognitive Training on Hyperactivity/Impulsivity Symptoms

Figure 30 Funnel Plots for Meta-Analyses of Effects of Cognitive Training on Global Executive Composite of BRIEF

Egger's test for small-study effects:

Regress standard normal deviate of intervention

effect estimate against its standard error

Number of studies = 13 Root MSE = 1.394

----------------------------------------------------------------------------------------------------------

Std_Eff | Coef. Std. Err. t P>|t| [95% Conf. Interval]

----------------------------------------------------------------------------------------------------------

slope | -.4808824 .4636719 -1.04 0.322 -1.501417 .5396524

bias | .5577286 1.673465 0.33 0.745 -3.125543 4.241

---------------------------------------------------------------------------------------------------------

Test of H0: no small-study effects P = 0.745

Figure 31 Egger’s Tests for Meta-Analyses of Effects of Cognitive Training on Global Executive Composite of BRIEF
